# Does the Type of Anaerobic Test Matter? A Comparison between the Anaerobic Intermittent Kick Test and Wingate Anaerobic Test in Taekwondo Athletes

**DOI:** 10.3390/sports10100154

**Published:** 2022-10-14

**Authors:** Stefanos Boutios, Alessandra di Cagno, Andrea Buonsenso, Marco Centorbi, Enzo Iuliano, Giuseppe Calcagno, Giovanni Fiorilli

**Affiliations:** 1 Department of Physical Education and Sport Science, National and Kapodistrian University of Athens, 15772 Athens, Greece; 2 Department of Motor, Human and Health Sciences, University of Rome “Foro Italico”, Lauro de Bosis Square 15, 00197 Rome, Italy; 3 Department of Medicine and Health Sciences, University of Molise, v. De Sanctis 1, 86100 Campobasso, Italy; 4 Faculty of Psychology, eCampus University, 22060 Novedrate, Italy

**Keywords:** martial art, mean and peak power, anaerobic capacity, blood lactate, functional evaluation, specificity

## Abstract

The specificity of training as well as the specificity of monitoring the training process are believed to be fundamental principles to efficiently plan and carry out the preparation and performance development of athletes. The Anaerobic Intermittent Kick Test (TAIKT) is a sport-specific field test used to specifically evaluate the anaerobic profile of Taekwondo athletes. The aim of this study was to verify whether TAIKT and the ‘gold standard’ Wingate Anaerobic test (WAnT) were both efficient means to optimally determine the anaerobic power and anaerobic capacity of Greek Taekwondo athletes at a middle-high technical level. Fifteen athletes, 10 females and 5 males (mean age 23.4 ± 4.14 years), underwent the two anaerobic tests (TAIKT and WAnT). The peak of power, the anaerobic capacity, and the peak of blood lactate (BL) were recorded. The two tests showed a moderate correlation with the *r* value ranging between 0.353 and 0.428, if applied to a sample of middle-high technical level athletes. Regarding the peak of BL, data indicated 40% concordance between the two tests with a coefficient of variation of 12%. Consequently, the two tests were correlated even if not interchangeable due to the different type of exercise required in these assessments. In conclusion, to assess the anaerobic performances and physiological characteristics of Taekwondo athletes, independently of their technical level, the WAnT resulted suitable, while to better assess the functional performance and specific demands of Taekwondo, the TAIKT is more indicated.

## 1. Introduction

The specificity of training as well as the specificity of monitoring the training process are believed to be fundamental principles to efficiently plan and carry out the preparation and performance development of athletes [1]. To elicit specific adaptations, a training programme must stress the metabolic systems that are engaged in performing the specific sport activity. Moreover, to enhance performance, both in terms of physical and technical skills, the involved muscle kinetic chains and the specific abilities of the specific sport must be trained and monitored [2,3].

The specificity of training influences all the stages of the planning of the training and characterizes the training from the beginning of the sport season [4]. Consequently, the monitoring process, which accompanies all training stages and allows to organize long and short-term programs, should be also as specific as possible in any phase.

In the Taekwondo performance model, athletes deal with brief periods of fighting activity-attacks (1–5 s) and non-fighting activity periods of rest of different duration, depending on the different Taekwondo specialities [5]. Aerobic and anaerobic power, muscular strength and power, flexibility, speed and agility are required in Taekwondo athletes [6]. Taekwondo athletes need high anaerobic power to perform fast movements in acceleration, such as punching and kicking [7]. Especially the high anaerobic power of the lower limbs appears to be conducive to achieving success in international competitions [8]. The mean anaerobic power generated by the athletes elicit high demands on the adenosine triphosphate (ATP)/creatine phosphate (CP) pathway but mainly on the glycolytic metabolic pathway [9]. Only moderate levels of cardio-respiratory fitness are required to support the metabolic demands of fighting and to support the recovery [8,9,10].

The common tool used to monitor the anaerobic power improvements is the ‘gold-standard’ 30 s Wingate anaerobic test (WAnT) [11], because it is suitable for different sports. The WAnT is a valid and reliable method of measuring maximal anaerobic power (peak and mean), the maximal anaerobic capacity as well as the reduction of power, known as anaerobic fatigue index, among Taekwondo elite athletes [8]. Previous studies highlighted that, during the WAnT, the ATP-PC pathway was about 28% and the glycolytic pathway was about 56%, while the aerobic pathway was only 16% of the energy contribution [12].

However, more specific Taekwondo-related tests are needed to better detect functional performance and specific demands of Taekwondo [10,13,14,15,16]. The Taekwondo Anaerobic Intermittent Kick Test (TAIKT) [16] is a sport-specific field test able to assess Taekwondo athletes’ anaerobic power (e.g., peak of power and mean of power expressed) based on the number of recorded kicks on an electronic body protector. Tayech et al. [16] stated that TAIKT’s physiological strain was comparable to that of a Taekwondo match of the same duration, considering the athletes during the test perform specific technical movements. In several studies referring to high level athletes’ anaerobic performances, the correlation between WAnT and TAIKT was found to be large to moderate.

The aim of the study was to verify if a specific test (TAIKT) and a gold standard test (WAnT) were both efficient to optimally determine the anaerobic power and anaerobic capacity of Taekwondo athletes of different technical levels. In this regard we assessed a concordance index to verify the possibility of using both tests in an interchanging manner.

## 2. Materials and Methods

### 2.1. Participants

Fifteen athletes (aged 23.4 ± 4.14), 10 females and 5 males, were recruited from different Taekwondo Greek clubs. All participants trained regularly with 3 sessions per week lasting approximately 90 min. The Taekwondo-technical level of recruited sample was from middle to high. The inclusion criterium was the regular training for at least 2 years and the exclusion criteria were (1) the presence of injuries in the previous 2 months, (2) the use of drugs that could influence the correct execution of the tests; (3) the presence of any situation, even temporary, that could have compromised the correct execution of the proposed procedures or the safety of the participants. Sample characteristics are shown in Table 1.

All participants and the parents of all the under-aged athletes gave their written informed consent, after a detailed illustration of the aims, benefits, and risks of the experimental procedures. The study was designed and conducted in accordance with the Declaration of Helsinki and approved by the bioethical local committee of the University of Molise (prot.11/2022).

### 2.2. Procedures

All participants underwent two testing sessions over two non-consecutive days in order to verify the efficiency of TAIKT, compared to the WAnT, in determining the anaerobic power and anaerobic capacity of competitive Taekwondo athletes. The tests were performed in a counterbalanced order: the participants were randomly divided into two groups: the first group performed TAIKT and then WAnT, while the second group performed WAnT and then TAIKT. Participants were required to avoid strenuous activities for at least 48 h before the testing session. The study was conducted in a controlled indoor environment at the same time of day (between 5 p.m. and 7 p.m.), to avoid the circadian rhythm influences, to avoid eating influences (more than three hours before each testing session), and under the same environmental conditions (i.e., temperature 25 °C and 63% humidity). The athletes wore their typical Taekwondo *dobok*.

Data were collected by researchers not involved in data analysis and during each assessment athletes and researchers could not provide or request information each other.

*Wingate anaerobic test (WAnT)*. The WAnT is a 30 s cycle-ergometer test developed to measure anaerobic power and capacity. Following a five-minute warm-up that included three sprints at varying resistances and a 3-min rest, the athlete started to pedal as fast as possible without any resistance [17]. Within three seconds, a fixed resistance was applied to the flywheel and the athlete continued to pedal at maximal effort for 30 s. At the end of the test, capillary blood lactate (BL) concentration was assessed using a portable BL analyser (Lactate Pro 2; Arkray, Kyoto, Japan). The fixed resistance applied to the flywheel was equal to the 7.5% of the athlete’s body weight [11]. During the test, participants were instructed to maximally accelerate from a seated posture to avoid the effect of postural changes. The participants were strongly and regularly encouraged throughout the test. The ergometer used for the test was a Monark 894E (Monark, Vansbro, Sweden). Knowing the resistance, the number of revolutions of the flywheel, the distance travelled, and the duration, it was possible to calculate power and work outputs. In particular, the power (in watts) was calculated for each 5 s interval and consequently 6 measurements were recorded at 5, 10, 15, 20, 25, and 30 s.

The following main 4 variables were collected for the statistical analysis: (1) *peak of BL*, assessed at the end of the test; (2) *peak of power*, namely the 5 s of the test during which the athletes produced the highest amount of power (generally the first 5 s of the test); (3) *mean of power*, namely the average power of the 6 assessments performed at 5, 10, 15, 20, 25, and 30 s; (4) *total anaerobic capacity*, namely the summation of the power produced during the 6 assessments; and (5) *fatigue index*, namely the percentual decrement in power calculated with the formula [Fatigue index = (Peak of power − lowest power)/peak of power × 100] [18].

*Taekwondo Anaerobic Intermittent Kick Test (TAIKT)*. The TAIKT required the athlete to perform the maximal number of stationary roundhouse kicks in a predetermined lapse of time, alternating right and left legs. Six sets of 5 s each were performed during the test, interspersed with 10 s of active recovery (i.e., bouncing movements) between each set. Consequently, the total duration was 30 s [16]. The kicks were executed on an electronic body protector (TK-Strike Protector; Daedo, Barcelona, Spain) placed around a punching bag, stabilized by a trainer. The number of kicks as well as the watt of each kick were automatically recorded on the computer. As with the WAnT, at the end of the test the capillary BL concentration was assessed using the same portable BL analyser. The participants were strongly and regularly encouraged to perform the test at maximal effort. Knowing the number of kicks performed in each set and the power of each kick, also in this case it was possible to calculate power and work outputs. In particular, the power (in watts) was calculated for each set summing the power of all the kicks thrown in that set.

Also for TAIKT, the following main 4 variables were collected for the statistical analysis: (1) *peak of BL*, assessed at the end of the test; (2) *peak of power*, namely the set in which the athletes produced the highest amount of power (generally the first set); (3) *mean of power*, namely the average of the power produced in the 6 sets; (4) *total anaerobic capacity*, namely the summation of the power produced in the 6 sets; and (5) *fatigue index*, namely the percentual decrement in power calculated with the formula [Fatigue index = (Peak of power − lowest power)/peak of power × 100] [18].

### 2.3. Statistical Analysis

Data was presented as means ± standard deviation (SD). Power analysis was performed *a priori*. Power analysis indicated that a sample size of 15 participants were necessary to achieve a power of 80% and a level of significance of 5% (two sided), for detecting a mean of the differences of 1.7 between pairs, assuming the standard deviation of the differences to be 2.1.

The peak of BL and the fatigue index obtained by the participants in the WAnT and TAIKT were compared using Pearson’s r correlation analysis. Furthermore, Lin’s concordance correlation coefficient (Lin’s CCC), Bland–Altman plot, and coefficient of variation (root mean square method) were also computed to evaluate the agreement between the measurements of the BL and fatigue index of the 2 anaerobic tests. Peak of BL and the fatigue index were also compared using Paired Student’s *t*-test in order to evaluate whether the results obtained in the WAnT and TAIKT resulted statistically significant.

Subsequently, the peak of power, the mean power and the total capacity expressed in the two tests were also analysed using Pearson’s correlation to evaluate whether a correlation existed between the external workloads assessed in the two tests. For the interpretation of the Pearson’s *r* results, the following ranges were used: values between 0 and 0.3 were considered as weak correlation; values between 0.3 and 0.7 were considered as moderate correlations; values between 0.7 and 1 were considered as strong correlations [19]. For the interpretation of the Lin’s CCC the following ranges were instead used: values < 0.90 were considered as poor agreement; values between 0.90 and 0.95 were considered as moderate agreement; values between 0.95 and 0.99 were considered as strong agreement; and values > 0.99 were considered as almost perfect [20].

All the analyses were performed using MedCalc Statistical Software version 18.2.1 (MedCalc Software, Ostend, Belgium).

## 3. Results

**Figure 1:**
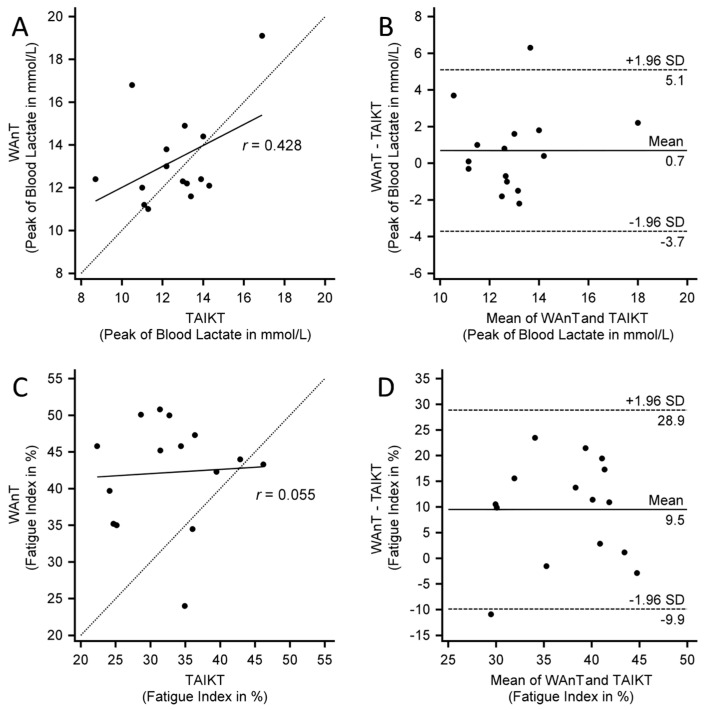
(**A**) shows the correlation plot between the peak of blood lactate measured in the WAnT (Wingate Anaerobic Test) and TAIKT (Taekwondo Anaerobic Intermittent Kick Test). The dotted line represents the function y = x, whereas the continuous line represents the correlation line. (**B**) shows the Bland–Altman plot between the peak of blood lactate measured in the WAnT and TAIKT. (**C**) shows the correlation plot between the fatigue index measured in the WAnT and TAIKT. (**D**) shows the Bland–Altman plot between the fatigue index measured in the 2 tests.

Descriptive data obtained from the 2 tests is reported in Table 2. The analysis performed on the peak of BL showed that the values obtained from WAnT and TAIKT had a moderate correlation (*r* = 0.428) whereas the Lin’s CCC resulted to be poor (CCC = 0.400). The coefficient of variation between the 2 tests relative to the assessment of the peak of BL was 12.5%. The analysis performed on the fatigue index showed instead that the values obtained from WAnT and TAIKT had a very weak correlation (*r* = 0.055) and the Lin’s CCC resulted to be very poor (CCC = 0.028). The coefficient of variation between the 2 tests relative to fatigue index was 26.3%. In Figure 1, the Bland-Altman plot, and the graph of correlation between the BL and the fatigue index obtained in the two tests are reported. Paired Student’s *t*-test showed that measures of peak of BL obtained from the two tests are not significantly different (*p* = 0.251), while the values of fatigue index obtained in WAnT are significantly higher in than those obtained in TAIKT (*p* = 0.002) (Table 2).

**Figure 2:**
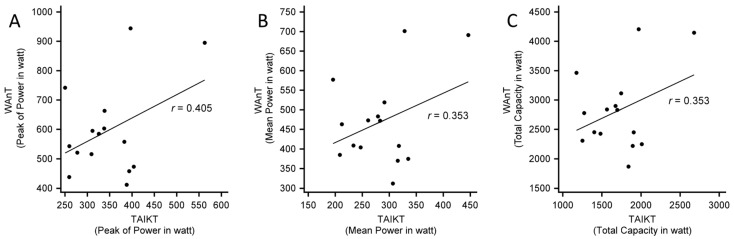
The figure shows the correlation plots between peak of power (**A**), the mean power (**B**), and the total anaerobic capacity (**C**) measured in the WAnT (Wingate Anaerobic Test) and TAIKT (Taekwondo Anaerobic Intermittent Kick Test).

The correlation of the peak of power, the mean power, and the total anaerobic capacity between the two tests resulted to be moderate with *r* values ranging between 0.353 and 0.405 (Figure 2).

## 4. Discussion

In the present study, two exhaustive tests both aimed at evaluating the anaerobic performance of Taekwondo athletes from medium to high level were compared: the ‘gold standard’ WAnT [11], which was an ergometry test based on a biking exercise, and the TAIKT [16], which required a succession of intermittent kicks (6 sets of 5 s each one) on an electronic body protector. It should be noted that both tests had the same time of execution (30 s) [7,21].

Since in another study the correlation among power and capacity variables and physiological characteristics (BL) was found to be moderate to large for high level athletes [21], the hypothesis was that the two tests resulted to be correlated for medium-high level athletes even with an underestimation or overestimation in all of the test variables.

The main result of the present study showed only a partial correlation between the gold-standard WAnT and the specific TAIKT. The results suggested that there was not a high magnitude of agreement between the two methods of evaluation and that athletes could have different performances in the two tests due to their different technical and physical preparation. Therefore, participants who had better results in the non-specific test frequently did not perform equally well in the specific test [22].

It is important to highlight that this finding did not indicate that one test was more valid than the other, but that the type of the exercise required to be carried out could affect assessments of anaerobic performance. The WAnT, where the athlete performs an incremental work on a bike and was encouraged to maximally accelerate from a seated position, allowed the athlete to reach her/his maximum effort. The TAIKT protocol required the athlete to reach the maximal frequency of stationary kicks. Despite kicks being a Taekwondo specific attack shot, the repetition of high intensity actions, alternating kicks on two legs, required an optimal technical preparation. Therefore, the TAIKT was more influenced by the technical level of the tested athletes [23]. In previous studies [16,24] the sample involved athletes of the Tunisian Taekwondo national team, while our sample involved athletes from medium to high technical level.

To monitor middle-high level athletes the two tests were both reliable tests but not interchangeable. TAIKT could be more indicated to assess the athlete’s high intensity and short duration specific abilities relevant to the Taekwondo, such as explosive kicks, and to verify the improvement of the athlete’s technical level, while the WAnT was useful to evaluate the anaerobic performance of the Taekwondo athlete using a “non-specific” procedure.

The moderate concordance involved both the BL levels and the anaerobic power/capacity outcomes. In relation to the peak of BL, data indicated 40% concordance between the two tests, with a coefficient of variation of 12%. The peak of BL, measured after an official Taekwondo tournament (close to 13.1 mmol/L), suggested that the competitive performance requires high lactic acidosis and that it was fundamental to monitor this variable [25]. In the TAIKT, our sample showed a mean lactate peak higher than those in other literature studies. The mean lactate peak recorded during the TAIKT (12.6 ± 1.9 mmol/L) was higher than those found by Tayech et al. [21] (11.0 ± 1.6 mmol/L). In the WAnT, the observed mean lactate peak value (13.3 ± 2.2 mmol/L) supported the correlation coefficient of *r* = 0.43 between the two tests.

Similar results were obtained in the measurements concerning peak power, which showed a moderate correlation (*r* = 0.405) between WAnT and TAIKT. Moderate-to-large correlation coefficients were observed by Tayech and colleagues [21], between the TAIKT and the WAnT (i.e., Peak of power *r* = 0.66 and Mean of power *r* = 0.62). In respect of the TAIKT, our measurements of the total wattage relative to the kicks performed in 5 s were coherent to those of Tayech [21], even if referring to the most powerful kick carried out during the 6 series of the test.

Probably in the TAIKT, as fatigue increased the coordination and balance were affected in performing alternated kicks with the two legs, and as a result the athletes of our study decreased the frequency of execution, showing an average power and BL value lower than those in the WAnT [26]. In the TAIKT, the athletes of international level, being not only physically fit but also technically well-trained, maintained an optimal kick frequency and consequently recorded mean power and BL values comparable to the results in the WAnT.

Regarding the total anaerobic capacity, the results confirmed the moderate correlation between the two tests, probably due to the different external work and work/rest ratio: the WAnT requires a continuous performance while TAIKT requires intermittent repetitive high intensity kicking.

The hypothesis that the moderate correlations between the two tests could be related to the different external work and work/rest ratio seemed to be supported by the very weak correlation in fatigue indexes obtained from both the two tests, indicating that the procedures induced different exhaustion level. In the WAnT, the athletes had a fatigue index ≈ 10% higher than in the TAIKT, suggesting that the continuous performance task of the WAnT induced a higher level of fatigue compared to the intermittent performance task of the TAIKT. This result was in accordance with the study of Tayech and colleagues [21], despite the data being not directly comparable as in Tayech’s study the fatigue index was reported in watt/s rather than in percentual values.

Probably the TAIKT resulted to be more accurate to provide information on the energy demands during Taekwondo combats, due to the similarity in the activity profiles and specific motor skills used in this assessment [27]. Kicks are a primary technique used in Taekwondo combats and, consequently, are relevant to evaluate the anaerobic profile of these athletes [28]. Different tests had different ability to describe anaerobic performance in Taekwondo athletes [29].

The limitations of the study are mainly related to the limited size of the sample and the fact that sex was not equally distributed in our samples (ten female and five male athletes). Moreover, the results were related to a specific technical level of Taekwondo athletes and, therefore, were not fully comparable with the few previous studies. Given the assumptions of this study, more researchers are needed for developing specific multidimensional tests.

## 5. Conclusions and Practical Application

This study showed a moderate correlation between WAnT and TAIKT if applied to a sample of middle-high technical level athletes, consequently the two tests do not appear interchangeable.

Our findings suggested that to assess the anaerobic performances and physiological characteristics of Taekwondo athletes, independently of their technical level, the WAnT was suitable, not being influenced by the Taekwondo-specific technical skills, while to better assess the functional performance and specific demands of Taekwondo, TAIKT was more indicated, especially for middle-high level athletes. These findings were influenced by the technical level of the athletes belonging to our sample. Therefore, to have a practical application of these data it is advisable to factor in differences in the preparation level among the subject samples.

## Figures and Tables

**Table 1:** Characteristics of the sample.

Participants (*n* = 15) 10 Females and 5 Males	Means ± SD (Males)	Means ± SD (Females)	Means ± SD (All)
Age (years)	21.4 ± 3.1	24.4 ± 4.4	23.4 ± 4.1
Weight (kilograms)	71.0 ± 13.2	62.6 ± 10.8	65.4 ± 11.9
Height (meters)	1.72 ± 0.06	1.70 ± 0.06	1.71 ± 0.06
Body Mass Index (kg/m^2^)	23.96 ± 4.45	21.63 ± 3.48	22.41 ± 3.84
Experience (years of competition)	15.80 ± 2.68	13.60 ± 6.02	14.33 ± 5.15

**Table 2:** Descriptive statistics of the results obtained from the 2 tests.

Outcomes	Wingate Anaerobic Test (WAnT)	Taekwondo Anaerobic Intermittent Kick Test (TAIKT)
	Means ± SD	Means ± SD
Peak of blood lactate (mmol/L)	13.28 ± 2.23	12.59 ± 1.94
Peak of power (watt)	596.40 ± 157.15	346.53 ± 80.21
Mean of power (watt)	469.47 ± 113.20	284.08 ± 63.65
Total capacity (watt)	2816.60 ± 679.77	1704.48 ± 381.91
Fatigue index (%)	42.2 ± 7.4 *	32.7 ± 7.0

* Significantly higher than TAIKT (*p* = 0.002).

## Data Availability

The data will be shared on reasonable request to the corresponding author.
